# Efficacy and Patient’s Acceptance of Alternative Methods for Caries Removal—A Systematic Review

**DOI:** 10.3390/jcm9113407

**Published:** 2020-10-23

**Authors:** Miguel Cardoso, Ana Coelho, Rui Lima, Inês Amaro, Anabela Paula, Carlos Miguel Marto, José Sousa, Gianrico Spagnuolo, Manuel Marques Ferreira, Eunice Carrilho

**Affiliations:** 1 Institute of Integrated Clinical Practice, Faculty of Medicine, University of Coimbra, 3000-354 Coimbra, Portugal; anasofiacoelho@gmail.com (A.C.); ruicarlosvaz@gmail.com (R.L.); ines.amaros@hotmail.com (I.A.); anabelabppaula@sapo.pt (A.P.); cmiguel.marto@uc.pt (C.M.M.); ze-93@hotmail.com (J.S.); eunicecarrilho@gmail.com (E.C.); 2 Institute of Biophysics, Faculty of Medicine, University of Coimbra, 3000-354 Coimbra, Portugal; 3 Institute of Experimental Pathology, Faculty of Medicine, University of Coimbra, 3000-354 Coimbra, Portugal; 4 Coimbra Institute for Clinical and Biomedical Research (iCBR), Area of Environment Genetics and Oncobiology (CIMAGO), Faculty of Medicine, University of Coimbra, 3000-354 Coimbra, Portugal; m.mferreira@netcabo.pt; 5 Clinical Academic Center of Coimbra (CACC), 3000-354 Coimbra, Portugal; 6 Department of Neurosciences, Reproductive and Odontostomatological Sciences, University of Naples “Federico II”, 80125 Napoli, Italy; gspagnuo@unina.it; 7 Institute of Dentistry, I. M. Sechenov First Moscow State Medical University, 119146 Moscow, Russia; 8 Institute of Endodontics, Faculty of Medicine, University of Coimbra, 3000-354 Coimbra, Portugal

**Keywords:** dental caries, dental caries treatment, alternative methods, conservative treatment, evidence-based dentistry

## Abstract

Dental caries is a disease of dental hard tissues, considered the most common non-communicable disease worldwide. Conventional treatments for caries removal are often associated with pain and fear, so different therapeutic approaches have been developed towards more conservative and pleasant treatments. This systematic review aimed to assess the efficacy and patient’s acceptance of alternative methods for caries removal compared to conventional methods. The Cochrane Library, Embase, Medline/Pubmed, Web of Science, and Clinical Trials databases were searched. Clinical trials of primary dental caries treated with alternative methods were included. The last search was performed on 5 August 2020. The Population, Intervention, Comparison, and Outcome (PICO) strategy was followed. Thirty-seven clinical trials were included, reporting caries removal using alternative (chemomechanical—Brix 3000, Carie-care, Carisolv and Papacarie, laser—Er:YAG (Erbium-doped Yttrium Aluminium Garnet) and Er,Cr:YSGG (Erbium, Chromium-doped Yttrium, Scandium, Gallium and Garnet), and a system combining air and sono-abrasion—Vector^®^ System) and conventional methods. Alternative methods tended to prolong treatment time and lessen anesthesia need. All treatments were effective in reducing cariogenic flora, and the restoration’s performance did not differ significantly. Chemomechanical solutions seemed to be the best option towards minimally invasive treatments, with good control during application and action and good treatment experiences for patients. Papacarie was demonstrated to be an effective method for caries removal with less pain, and superior acceptance by patients when compared to conventional treatments.

## 1. Introduction

Dental caries is a disease of dental hard tissues, being the most common chronic disease amongst all oral conditions [1,2,3,4,5]. Its onset and spread varies with geographic location and socioeconomic status, the untreated decay tending to increase with decreasing income [1,6]. According to the World Health Organization (WHO), dental caries is the most common non-communicable disease worldwide [7]. Dental decay has been related to multifactorial etiology, where diet and oral flora play an essential role, being the acid production by microbial metabolism of sugars and the prolonged periods of low oral pH, the critical factors for tooth demineralization. It starts with small surface roughness or sub-surface demineralization, progressing to cavitation [1,5,6,8,9,10,11]. Then, if untreated, it may cause pulp involvement, swelling, abscess, and systemic signs and symptoms [4,8].

Conventional cavity preparation and caries removal methods use mechanical means, mostly burs, and are often associated with pain and fear, especially for children [5,12,13]. Although the pain can be managed through local anesthesia, fear of the needle, noise, and vibration of mechanical preparation remains a cause of discomfort for the patient [5,10,13,14]. Moreover, these techniques present the risk of easily removing healthy dental tissues or damaging the pulp through temperature rise, which may be in the origin of discomfort (thermal stimulation) [5,10].

Until today, different therapeutic approaches regarding dental cavities have been discussed and reconsidered in order to be as conservative as possible and to preserve tooth structure by only removing the irreversibly damaged dental tissues [12,13,15,16]. This leads to increased tooth longevity and prevents the repetitive restorative cycle [12,15]. Although mechanical methods for caries removal are widely accepted quick techniques, various alternative therapeutic approaches have been demonstrated to be promising, such as chemo-mechanical methods, lasers, or sono/air abrasion [10,12].

Chemo-mechanical caries removal systems are solutions which act on the principle of carious tissue softening to facilitate their removal and apply sodium hypochlorite (NaOCl)- or enzyme-based agents [17,18,19]. After use, the gel often changes color and becomes turbid or produces bubbles, making easier the identification of the occurring reaction, completion or absence (meaning no remaining decayed tissue); then, the softened tissue is removed by non-cutting tip instruments [17,18,19]. The enzyme-based materials can be associated with anti-inflammatory properties, which can lead to better treatment experiences and less induced pain. Agents with hypochlorite are also associated with less necessary anesthesia since the sodium hypochlorite has its action within the already damaged collagen fibrils [18,19]. Examples of NaOCl-based agents are GK-101E (Caridex, National Patent Medical Products, N.B., USA) and Carisolv (Mediteam, Sweden); examples of enzyme-based agents are Carie-care (Uni-Biotech Pharmaceuticals Private Limited, Chennai, India), Papacarie (F&A Laboratório Farmacêutico, São Paulo, Brazil), and Brix 3000 (Brix S.R.L., Argentina).

Laser ablation systems for caries removal use laser wavelengths that achieve a major interaction with either the mineral or the water content or both, in the decayed tissues (unless there is plasma-mediated ablation by ultrashort pulses). In the most common systems for caries removal, the primary mechanism of action is the water heating at the surface and the subsurface which causes its expansion, and the tissue is exploded out from the surface [19,20,21]. The most commonly used laser systems for caries removal are the Er:YAG (Erbium-doped Yttrium Aluminium Garnet) laser and the Er, Cr: YSGG (Erbium, Chromium-doped Yttrium, Scandium, Gallium and Garnet) laser.

Air-abrasion systems for caries removal are a technique based on the blasting of the tooth surface with high-velocity particles carried in a stream of air, removing tissue from the cavity. Furthermore, sono-abrasion systems for caries removal use high frequency, sonic, air-scalers with modified abrasive tips; different shaped tips help prepare the intended cavity outlines and remove carious dentin [19,22].

The purpose of this study was to carry out a systematic review of the literature to answer the Population, Intervention, Comparison, and Outcome (PICO) question: “In removing dental caries from deciduous and permanent decayed teeth, how efficient are alternative methods for caries removal, comparing to the conventional mechanical methods?” (Table 1).

## 2. Materials and Methods

The protocol was registered in the International Prospective Register of Systematic Reviews (PROSPERO, www.crd.york.ac.uk/prospero) with the number CRD42020157372.

The present systematic review was performed following the recommendations of Preferred Reporting Items for Systematic Reviews (PRISMA) [23] guidelines, the A MeaSurement Tool to Access Systematic Reviews (AMSTAR) instrument [24] and the Cochrane Handbook of Systematic Reviews of Interventions [25] version 6.

### 2.1. Eligibility Criteria

#### 2.1.1. Types of Studies

Only Controlled Trials were analyzed. No restrictions on the region, year of publication, language, or publication status were considered.

#### 2.1.2. Types of Participants

Children or healthy adult patients with a clinical diagnosis of primary dental caries were considered for inclusion. Only cavitated dentin lesions were considered, and white spot lesions and fissure lesions were excluded. Studies on patients from specific study populations (like special care needs or syndromic) were also excluded. Studies applying questionnaires to access previous treatment experiences with alternative methods for caries removal were excluded from consideration. Only teeth without clinical and radiographic signs of pulpal damage were included. A schematic representation of the inclusion and exclusion criteria is presented in Table 2.

#### 2.1.3. Types of Interventions

(1) Dental caries treatment with conventional mechanical methods (control), using high-speed rotary instruments, excavators, or both.
(2) Dental caries treatment with chemomechanical methods, comprising Brix 3000, Carie-Care, Carisolv or Papacarie, in combination with blunt excavators.
(3) Dental caries treatment with laser systems, comprising Er:YAG or Er,Cr:YSGG lasers.
(4) Dental caries treatment with air- and/or sono-abrasion systems, using the Vector System (VS, Duerr Dental, Bietigheim-Bissingen, Germany).

The comparisons intended were: chemomechanical versus control, laser versus control, VS versus control, the different solutions from the chemomechanical methods from each other, the Er:YAG laser versus Er,Cr:YSGG laser, and between all the alternative methods.

#### 2.1.4. Types of Outcome Measures

##### Primary Outcomes

Dental caries removal efficacy assessed through treatment time, the evaluation of remaining caries, cavity dimensions or DIAGNOdent (Kavo, Germany) measures, the anesthesia required for treatment, and the colony-forming units (CFU) count measurement.

##### Secondary Outcomes

Restorations performance during the reported follow-up periods.

Patient’s pain perception/behavior during treatment (all methods/scales of clinical assessment were considered, if applied immediately, before, during, or after treatment).

No restrictions on the length of follow-up were considered.

### 2.2. Search Strategy

#### 2.2.1. Electronic Search

For the identification of studies for this review, an electronic search was performed using the databases: Cochrane Library (www.cochranelibrary.com), Embase (www.embase.com), Medline/PubMed (www.ncbi.nlm.nih.gov/pubmed), Web of Science (www.webofknowledge.com), and Clinical Trials database (www.clinicaltrials.gov). The search strategy was firstly developed for Medline/PubMed using a combination of controlled vocabulary and free text terms, and then appropriately revised for each database. The search formulas are described in detail in Table 3, according to each searched database.

No restrictions on language or date of publication during the electronic databases screening were applied. The last literature search was performed on 5 August 2020. When the articles of interest were not available online, an attempt to contact the authors was made.

#### 2.2.2. Searching Other Sources

The reference lists of the relevant articles were manually searched to explore additional studies.

### 2.3. Data Collection, Extraction, and Analysis

#### 2.3.1. Selection of Studies

The titles and abstracts retrieved were downloaded to EndNote (X9.3.3, Clarivate Analytics, Philadelphia, PA, USA) software. Two review authors carried out the analysis and selection of papers and decisions about eligibility independently, in duplicate. Any disagreement was discussed, and the opinion of a third reviewer was sought out when necessary.

#### 2.3.2. Data Extraction and Management

For each included study, descriptive and quantitative information was collected independently, in duplicate, by two review authors, and included:

General study characteristics: authors and year of publication

Specific trial characteristics: population (deciduous or permanent dentition and number of treated lesions) and interventions

Primary outcomes: treatment time, caries removal, patient’s request for anesthesia and CFU count in dentin

Secondary outcomes: restorations and the patient´s pain perception/behavior during treatment. All follow-up periods and respective clinical evaluation of interest were reported

#### 2.3.3. Assessment of Risk of Bias in the Included Studies

The clinical trials’ risk of bias was assessed using the Bias Risk Assessment Tool described in the Cochrane Handbook of Systematic Reviews of Interventions [25] by two independent reviewers. It comprises the randomization, allocation concealment, blinding of participants and personnel, blinding of examiners, completeness of outcome reporting, selective outcome reporting, and other potential biases.

Regarding the identification of other biases, potential threats to the studies’ validity included: the differential extent of previous experiences regarding dental caries treatment and the previous exposure to different treatment options, the different anesthesia protocols or medication given previously to the intervention between control and test groups, patient’s experience or behavior during treatment, the performance of treatment on the same day (cumulative stress effect) or separately in time (different mind state), or the variation of tip positioning (laser systems).

The study’s risk of bias was used in sensitivity analysis to test the conclusion’s robustness, but it was not used to exclude studies from the present review. To examine the overall risk of bias for each study, the authors used all the domains of risk of bias. If all domains were at low risk, the study was deemed to be at low risk of bias. If any domains had an unclear risk, then the study was classified as having an unclear risk of bias; however, if one or more domains were assessed as being at a high risk of bias, so was the study.

## 3. Results

### 3.1. Search Results

**Figure 1:**
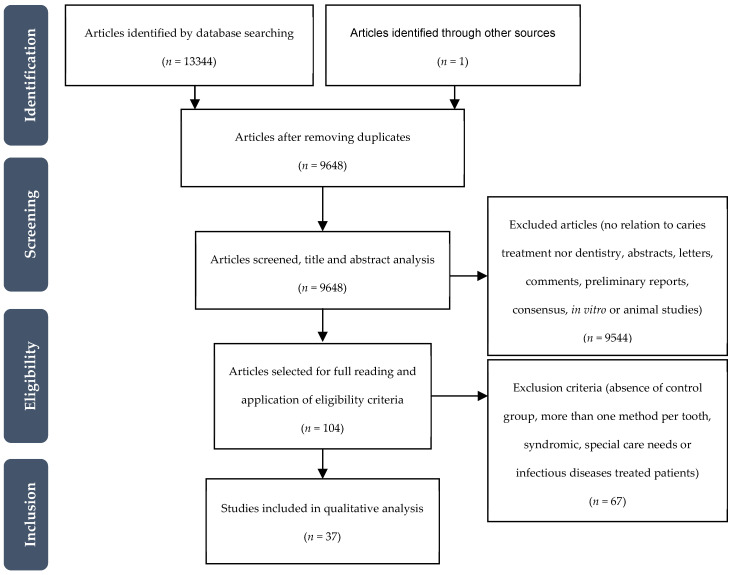
Flowchart of the studies selection process.

The initial search resulted in 13,344 references: 2849 from Cochrane Library, 3002 from Embase, 2831 from Medline/Pubmed, 4613 from Web of Science, and 49 from the Clinical Trials database. After removing the duplicates, the remaining record’s titles and abstracts were screened, and 104 relevant studies were obtained. After full-text analysis, 36 references were included in this systematic review. One additional study was included from the references list analysis of the already included studies (Figure 1).

### 3.2. Included Studies

#### 3.2.1. Design

Two [26,27] included studies followed a cross-over trial design, while eighteen [10,28,29,30,31,32,33,34,35,36,37,38,39,40,41,42,43,44] had a split-mouth design. One study [45] followed a two parallel-arms trial, five studies [46,47,48,49,50] provided one treatment per patient while five studies [15,51,52,53,54] had one or more treatment per patient. In six studies [12,55,56,57,58,59] it was not possible to identify the study design, or the provided information was not enough to qualify them.

#### 3.2.2. Participants

In total, about 1600 patients with primary dental caries were treated in the included studies. The treated patients’ age ranged from 3 [28,30] to 84 [51] years, with an overall mean of 10 years One study [52] did not specify the included patients’ age range, mentioning only that they were older than 18 years. In one study [55], only the total number of patients was mentioned. In two included studies [57,58], the number of treated caries in each group was not reported.

#### 3.2.3. Interventions

Five studies included more than one comparison [15,41,49,54,56]. The comparisons included in the trials were:

Chemomechanical versus Control [12,26,27,29,30,33,34,35,36,37,38,43,45,48,50,51,53,59]

Laser versus Control [10,28,31,32,39,40,42,44,46,47,52,57,58]

VS versus Control [55]

Carisolv versus Papacarie versus Control [15,41,54]

Carisolv versus Papacarie versus Er:YAG laser versus Control [56]

Sodium hypochlorite gel versus Brix 3000 versus Control [49]

#### 3.2.4. Outcomes

The outcomes provided by each included study are reported in Table 4, Table 5 and Table 6, presenting studies comparing one chemomechanical solution to conventional treatments, studies comparing a laser system to mechanical treatments and for studies having more than one alternative treatment approach, respectively. A summary of the relevant findings is presented in Table 7.

Twenty-three studies [12,15,26,27,29,30,33,34,35,36,37,38,41,43,45,48,49,50,51,53,54,56,59] reporting chemo-mechanical caries removal systems were included. Two [43,49] involved the use of Brix 3000, three [35,36,50] the use of Carie-care, nine [15,26,29,41,48,51,53,54,56] the use of Carisolv and thirteen [12,15,27,30,33,34,37,38,41,45,54,56,59] the use of Papacarie.

Thirteen included studies [10,28,31,32,39,40,42,44,46,47,52,57,58] showed laser caries removal systems: two studies [31,52] involved the use of the Er,Cr:YSGG laser system and 11 [10,28,32,39,40,42,44,46,47,56,57,58] the use of the Er:YAG laser system.

Regarding air—or sono-abrasion caries removal systems, only one study [55] was included and analyzed, which evaluated the use of the Vector^®^ System.

#### 3.2.5. Risk of Bias in the Included Studies

**Figure 2:**
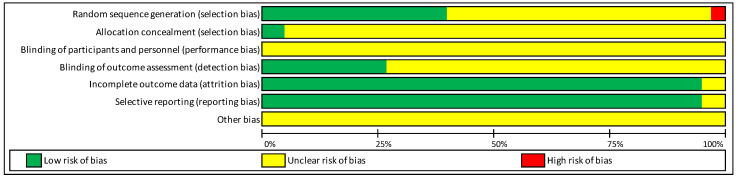
Schematic representation of the included studies methodological quality assessment.

The included studies’ quality assessment results are presented in Table A1 (Appendix A) and are schematically represented in Figure 2.

*Randomization*

Only one [31] of the included studies presented evident flaws in the methodological description of random sequence generation (the treatment was decided based on the number of the tooth), while 56.76% of the papers presented insufficient information to assess the selection bias and 40.54% described randomization procedures with low risk of bias.

*Allocation*

The allocation concealment was not clear in 94.6% of the studies. Only two studies [10,45] were considered as having a low risk of selection bias in this criterion, where proper envelopes were used.

*Blinding*

None of the studies provided enough data to clarify if the blinding of the participants and personnel (performance bias) was performed or not.

The blinding of the outcome assessment (detection bias) was performed in 27.03% of the included studies. The remaining studies (72.97%) were considered to have an uncertain risk of performance and detection bias since lacking information concerning these criteria.

*Incomplete outcome data*

Only two studies [46,47] were found to have incomplete outcome data (5.40% of attrition bias).

*Selective reporting*

Two studies [46,47] were found to have selective reporting (5.40% of reporting bias).

*Other biases*

All studies were considered to have an uncertain risk related to other biases, where the criteria previously stated were considered to interfere with the outcomes assessed. In addition, the generally synthetized reports of the methodology in most of the included studies led to uncertainty towards the interference of additional biases.

The performance of treatment under different field isolation conditions was considered to reduce the robustness of the comparisons made between studies, and was, therefore, considered in the analysis of the data.

Overall risk of bias

None of the studies scored a low overall risk of bias since none of the included studies had all the criteria classified as having a low risk. One study [31] scored a high overall risk of bias because of clear flaws in the groups’ random sequence generation. The rest of the studies had an unclear overall risk of bias because there were not enough data regarding the methodology to allow a complete evaluation of several criteria in the used bias assessment tool.

#### 3.2.6. Effects of Interventions

##### Chemomechanical versus Control

Regarding this intervention, one study [43] involved the use of Brix 3000, three studies [35,36,50] the use of Carie-care, five studies [26,29,48,51,53] the use of Carisolv, and nine [12,27,30,33,34,37,38,45,59] the use of Papacarie. All these studies had an overall unclear risk of bias.

*Treatment time*

All the fourteen included studies, all alternative treatment approaches demonstrated longer treatment time. Specifically, this aspect was reported in one study using Brix 3000 [43], in two studies that involved Carie-care [35,50], in three using Carisolv [26,48,51] and in two studies evaluating Papacarie [12,45].

*Caries removal (remaining caries, cavity dimensions, or other)*

Six studies reported caries removal related outcomes. 

The final cavity of the conventionally treated tooth was wider. Carisolv produced significantly smaller free caries lesions in one [26] out of three studies and Papacarie [12] in one out of one study.

Regarding efficacy in caries removal and considering the several and different criteria described in the included studies, there was no statistical difference between effectiveness of caries removal with rotary instruments and Carie-care in one [35] out one study and Carisolv in two [48,53] out two studies. However, in one study [48], Carisolv was statistically more efficient than the excavator.

*Request for anesthesia*

In the five studies starting treatment without anesthesia, patients receiving conventional treatments requested anesthesia more often than treatment with Carisolv [26,51] and with Papacarie [34,37,38].

*CFU count*

Five studies reported CFU dentin count after treatment, showing significantly reduced total bacterial count. However, although two studies [27,29] reported similar reductions after conventional treatments and treatment with Carisolv and Papacarie, three studies reported higher reductions when using alternative approaches, such as Brix 3000 [43] and Papacarie [12,38].

*Restorations performance*

In the six included studies [30,33,36,37,51,59] performing restoration assessment during follow-up periods, there were no significant differences regarding the restoration´s success between methods.

*Patients pain perception/behavior*

Fourteen studies [12,26,27,33,34,35,36,43,45,48,50,51,53,59] assessed the patients pain perception or behavior during the intervention. Patients receiving alternative approaches for caries lesions showed significantly better treatment experiences and fewer signs of discomfort or pain during the consultation in ten studies [12,26,27,34,35,43,45,48,50,51]. Only one study [33] revealed similar pain experience between groups. The remaining studies [36,53,59] reported a more positive treatment experience with alternative methods, but without significant differences.

##### Laser versus Control

Regarding this intervention, eleven studies [10,28,32,39,40,42,44,46,47,57,58] involved the use of the Er:YAG laser and two studies [31,52] the Er,Cr:YSGG laser system. One study [31] had an overall high risk of bias, and the others [10,28,32,39,40,42,44,46,47,52,57,58] had an overall unclear risk of bias.

*Treatment time*

Treatment with the Er:YAG laser was significantly longer than the conventional treatments in four [10,28,32,42] out of five studies measuring treatment time, while the Er,Cr:YSGG laser did not significantly increase treatment time [31].

*Caries removal (remaining caries, cavity dimensions, or other)*

In two studies [46,47] using the Er:YAG laser, the energy needed for caries treatment in the deciduous dentition was significantly less than that required for the permanent dentition.

*Request for anesthesia*

Patients receiving treatment with the Er:YAG laser requested less anesthesia than those treated with conventional methods in two studies [10,46] and significantly less anesthesia was requested in one study [47].

*CFU count*

None of the included studies reported the CFU count in dentin.

*Restorations performance*

In the five studies [10,40,42,44,52] assessing restorations performance during follow-up periods, there were no significant differences between methods.

*Patients pain perception/behavior*

Twelve studies assessed the patient’s pain perception or behavior during the intervention. Patients receiving treatment with a laser showed significantly better treatment experiences and fewer signs of discomfort or pain during the consultation in seven studies [10,28,32,39,52,57,58]. In one study [57], the smell and taste complaints were significantly higher in the patients receiving treatment with the Er:YAG laser.

##### VS versus Control

One study [55] was included regarding this intervention, which has an overall unclear risk of bias. This study evaluated the use of the Vector^®^ System (test group, *n* = 14), which is a method combining both ultrasonic effect and micro-abrasive action, in comparison to bur treatment (control group, *n* = 13). The mean treatment time was 5.5 and 16.8 min for the control and test group, respectively. Treatment in the control group was faster (*p* < 0.001). A verbal pain scale was used to assess the patient’s pain perception, where the control group showed higher scores, namely more pain during treatment, comparing to the test group (*p* < 0.001); girls demonstrated higher scores among all study patients (*p* < 0.01).

*Treatment time*

Treatment with VS was significantly longer than conventional treatment.

*Caries removal (remaining caries, cavity dimensions, or other)*

The included study did not report remaining caries or the cavity dimensions between VS and the Control treatment.

*Request for anesthesia*

The anesthesia requested by the patients during treatment was not reported in the included study.

*CFU count*

The included study did not report the CFU count in dentin.

*Restorations performance*

The performance of the restorations was not reported in the included study.

*Patients pain perception/behavior*

Treatment with VS induced significantly less pain, and girls showed significantly higher pain scores among the study population.

##### Carisolv versus Papacarie versus Control

Regarding this intervention, three studies [15,41,54] were included. All these studies had an overall unclear risk of bias.

*Treatment time*

Carisolv and Papacarie showed longer treatment times. However, Papacarie was faster than Carisolv in one study [41] and significantly quicker than Carisolv in another study [54].

*Caries removal (remaining caries, cavity dimensions, or other)*

In one [41] of the included studies, Papacarie was significantly more efficient than Carisolv within the criteria used. In another study [54], there were less remaining caries in the Papacarie group than the Carisolv treated patients.

*Request for anesthesia*

None of the included studies reported the anesthesia requested by the patients during treatment.

*CFU count*

In one [15] of the included studies, both conventional and alternative methods significantly reduced dentin bacterial count with no differences identified between approaches.

*Restorations performance*

None of the included studies assessed the restoration´s performance.

*Patients pain perception/behavior*

In two of the included studies [41,54], Papacarie induced significantly less pain and offered a more comfortable treatment approach, being the most accepted.

##### Carisolv versus Papacarie versus Er: YAG Laser versus Control

One study [56] was included regarding this intervention, which has an overall unclear risk of bias.

*Treatment time*

Treatment with the Er:YAG laser was significantly faster than treatment with Carisolv and Papacarie. Treatment with Papacarie was slightly quicker than Carisolv.

*Caries removal (remaining caries, cavity dimensions or other)*

The included study performed measurements with DIAGNOdent, where the laser-treated teeth showed the highest percentage of change after treatment, that was significantly higher than that measured in teeth treated with Carisolv and Papacarie.

*Request for anesthesia*

The included study did not report the anesthesia requested by the patients during treatment.

*CFU count*

The CFU count was not performed in the included study.

*Restorations performance*

There was no assessment of the restoration’s performance during follow-up periods.

*Patients pain perception/behavior*

All the alternative methods were significantly less painful than the conventional method.

##### Sodium Hypochlorite Gel versus Brix 3000 versus Control

One study [49] was included regarding this intervention, which has an overall unclear risk of bias.

*Treatment time*

Treatment with the sodium hypochlorite gel and Brix 3000 was significantly longer than that of conventional methods.

*Caries removal (remaining caries, cavity dimensions or other)*

No outcomes related to caries removal were reported in the included study.

*Request for anesthesia*

The included study did not report the anesthesia requested by the patients during treatment.

*CFU count*

The CFU count was not performed in the included study.

*Restorations performance*

There was no assessment of the restoration´s performance during follow-up periods. 

*Patients pain perception/behavior*

Treatment with Brix 3000 was significantly less painful than the conventional methods, followed by treatment with the sodium hypochlorite gel.

## 4. Discussion

### 4.1. Summary of Main Findings

Despite representing prolonged treatment times, alternative methods may be preferable, allowing more conservative treatments, being more selective in removing decayed tissue and preserving more healthy tissue. The chemomechanical agents may be the most conservative treatment approach between the three modalities of alternative treatments because of their specific action towards decayed dentin. In addition, laser and air- or sono-abrasion methods present the disadvantage of no sensory feedback to the clinician during treatment, making it impossible to immediately evaluate the extent of caries removal.

Fear and anxiety remain two common aspects of dental treatments, the rotary instruments being one of their greatest triggers [60,61,62]. Alternative methods for caries removal showed a tendency to produce more comfortable treatment experiences and a higher acceptance and preference towards future treatments. Sometimes, the smell and taste were pointed out as negative factors when using alternative methods. However, vibration, sound, and discomfort induced by mechanical approaches were considered a more unpleasant treatment experience and were, especially in younger patients, the triggers for a more uncooperative behavior during treatment.

Although the request for anesthesia is subjective to the patients’ fear towards the intervention and possibly the existence of previous traumatic experiences with conventional techniques, leading to the preventive request of anesthesia to avoid unwanted discomfort, alternative methods induced pain less frequently [63,64]. However, it is important to evaluate the extension of the caries, their progression and the pulpal response before the intervention, as well as the patients willing towards anesthesia.

In the chemomechanical treatments, despite every method inducing less pain in patients compared to mechanical treatment, it is important to acknowledge the tendency for statistically significantly less pain reported when using the enzyme-based agents, as Carie-Care [35,50], Papacarie [12,27,34,41,45,54] and Brix 3000 [43,49]. This may occur due to the bactericidal and anti-inflammatory action of the included enzymes [17,19]. Both laser systems, Er:YAG [10,28,32,39,57,58] and Er,Cr:YSGG [52], induced significantly less pain compared to the mechanical methods. However, smell and taste were found to be factors for increased anxiety [32,57]. Vector System also presented with significantly less induced pain, and girls were more likely to report pain.

The treatment experience was better with alternative methods. Additionally, when necessary, a proper and careful anesthetic administration, the use of a rubber dam, the reduction of anxiety factors, and an overall well managed pain (when existing) during treatment will improve the experience and the patient’s behavior, especially in children. In these conditions, chemomechanical are the easiest to control and the preferable method for more conservative approaches. Further studies are needed to more accurately define wavelengths of laser systems and air- or sono-abrasion systems to assess the use of less harmful abrasive elements for healthy tissues. Moreover, improvement of study designs and their report is needed to extract more accurate conclusions.

The restorations performed by each method did not significantly differ from each other in terms of longevity and survival. Further discussion in this matter is not possible because of the differences between the clinical restoration protocols ranging from a wide variety in the isolation of the operative field (cotton rolls versus rubber damn), the restorative materials, the restoration criteria evaluation, and the follow-up times of each included study.

### 4.2. Overall Completeness and Applicability of Evidence

This review’s objective was to assess the efficacy of caries removal when using alternative methods compared to the conventional ones. From the extensive analysis of the extracted data, every method for caries removal seems effective and induces less pain than traditional mechanical methods. In the chemomechanical solutions, enzyme-based agents present better results, especially related to patient´s perception, experience, and acceptance. Papacarie seems to be the more adequate solution; however, this may be due to its use in most of the included studies regarding chemomechanical treatments. Laser systems are also interesting treatment options in achieving the caries removal and inducing less pain. However, as mentioned before, it comes with some disadvantages to the treatment experience. Air- and sono-abrasion are promising methods but require more studies.

In terms of caries removal efficacy, there was a wide variety of efficiency in criteria assessment. It was carried out either by a dental explorer examination or the application of caries detector dye. The first is examiner dependent, and thus is subjective. The latter, stains not only the outer infected tissue, but also the demineralized inner zone of caries, less mineralized dentin, and at the enamel–dentin junction, which also makes it not a very good indicator of bacterial invasion of the tissues and may lead to overpreparation. The use of laser detection devices, like DIAGNOdent, have been suggested as more adequate instruments to ensure the complete removal of infected dentin [65,66].

### 4.3. Quality of Evidence

Regarding the quality assessment of all included studies, the performance bias was common since the characteristics of the caries removal systems at use are easily distinguished from each other (conventional versus alternative systems). In general, the studies were found to present insufficient information on their methodology. On other biases, previous experiences were considered because alternative methods for caries removal are not the standard first-line treatment, and patients were certainly treated in previous sessions with rotary instruments. Traumatic events may have occurred, influencing the reported treatment experience with different methods. Additionally, the patient´s state of mind influences their behavior and reported experience, and it may bias the results because of its subjective nature or the comparison of patients with different exposures where the diverse backgrounds of each patient will bias the results.

### 4.4. Potential Biases in the Review Process

A sensitive search of multiple databases was conducted to identify the potential studies to be included in this review, where no restrictions on language were applied. An attempt to contact some study authors was made for missing information, however, without success. The authors recognize that the missing data and the included studies’ bias reduces the robustness of the analysis performed. Still, there was consistency in the workflow, and the reasons behind the conclusions are reported.

## 5. Conclusions

### 5.1. Implications for Practice

Alternative methods for caries removal tend to prolong treatment time and cause fewer requests for anesthesia during treatment; however, dentition, cavity extension, and pulpal response before treatment and patient-related factors should be considered when establishing the treatment plan. 

Both conventional and alternative approaches are efficient in reducing cariogenic flora from the cavities. The marginal integrity of restorations did not prove to differ significantly between methods for caries removal.

Patients reported more pleasant treatment experiences with alternative treatment approaches and higher percentages for acceptance and preference in future treatments for alternative methods were registered.

Chemomechanical solutions seem to be the best option for minimally invasive treatments, with good control of their application and action, as well as good treatment experiences for patients. Papacarie was the most studied solution in this treatment modality and presented efficiency for caries removal and high patients’ acceptance.

### 5.2. Implications for Research

More studies are needed, comparing more than one alternative treatment simultaneously. Furthermore, nowadays, standards in restorative dentistry ought to be followed to perform treatments with better quality and long-lasting restorations.

The studies’ methodology should be completely reported. Poorly written clinical studies do not present enough data to allow the readers to reasonably analyze the study and apply their conclusions in daily clinical practice.

## Figures and Tables

**Table 1:** Population, Intervention, Comparison, and Outcome (PICO) question.

Criteria	Description
P (population)	Deciduous and permanent decayed teeth
I (intervention)	Alternative caries removal (chemomechanical methods, laser, sono-/air-abrasion)
C (comparison)	Conventional mechanical methods for caries removal (rotary or hand instruments)
O (outcome)	Efficacy for caries removal (time for treatment, caries removal, anesthesia and colony forming units count)

**Table 2:** Inclusion and exclusion criteria.

Inclusion criteria	Controlled trials
	Primary caries lesion(s) treatment in healthy patients
	Caries lesion on primary or permanent dentition
	Cavitated dentin lesions
	One treatment per tooth
Exclusion criteria	Abstracts, letters, comments, preliminary reports, consensus recommendations, in vitro or animal studies
	Secondary caries lesion
	More than one technique applied per tooth, including the opening of the cavity with a different technique from that of caries removal
	Atraumatic restorative treatment (with no complete removal of decayed tissue), hall technique, non-restorative caries treatment, and related treatment approaches
	Specific populations (special care needs, infectious diseases, syndromic patients)

**Table 3:** Research strategy used.

Database	Search Strategy
Cochrane Library	#1 MeSH descriptor: [Dental Caries] explode all trees
	#2 carie*
	#3 cavit*
	#4 decay
	#5 #1 OR #2 OR #3 OR #4
	#6 alternative
	#7 clean*
	#8 removal
	#9 laser
	#10 Er-YAG
	#11 Er:YAG
	#12 GK-101*
	#13 NMG
	#14 papain
	#15 brix 3000
	#16 biosolv
	#17 solvent*
	#18 carie-care
	#19 papacarie
	#20 carisolv
	#21 *n*-monochloroglycine
	#22 caridex
	#23 ozone
	#24 “sono abrasion”
	#25 “air polishing”
	#26 photo-ablative
	#27 enzyme*
	#28 chloramine
	#29 “toluidine blue”
	#30 #6 OR #7 OR #8 OR #9 OR #10 OR #12 OR #13 OR #14 OR #15 OR #16 OR #17 OR #18 OR #19 OR #20 OR #21 OR #22 OR #23 OR #24 OR #25 OR #26 OR #27 OR #28 OR #29
	#31 #5 AND #30
Embase	(cavit* OR decay OR carie* OR ‘dental caries’/exp OR ‘dental caries’) AND ((removal OR clean* OR alternative OR ‘laser’ OR ‘laser’/exp OR laser OR ‘er yag’ OR ‘er:yag’ OR ‘gk 101*’ OR nmg OR ‘papain’ OR ‘papain’/exp OR papain OR brix) AND 3000 OR biosolv OR solvent* OR ‘carie care’ OR ‘papacarie’ OR ‘papacarie’/exp OR papacarie OR ‘carisolv’ OR ‘carisolv’/exp OR carisolv OR ‘*n-*monochloroglycine’ OR caridex OR ‘ozone’ OR ‘ozone’/exp OR ozone OR ‘sono abrasion’ OR ‘air abrasion’ OR ‘air polishing’ OR ‘photo ablative’ OR enzyme* OR ‘chloramine’ OR ‘chloramine’/exp OR chloramine OR ‘toluidine blue’/exp OR ‘toluidine blue’) AND (humans)/lim AND (clinical study)/lim
Medline/Pubmed	((((cavit* OR decay OR carie* OR “Dental Caries”(Mesh)) AND (removal OR clean* OR alternative OR laser OR Er-YAG OR ER:YAG OR GK-101* OR NMG OR papain OR brix 3000 OR biosolv OR solvent* OR carie-care OR papacarie OR carisolv OR *n-*monochloroglycine OR caridex OR ozone OR “sono abrasion” OR “air abrasion” OR “air polishing” OR photo-ablative OR enzyme* OR chloramine OR “toluidine blue”)))) AND Clinical Trial(ptyp))
Web of Science	(TS = (“dental decay” OR “tooth decay” OR carie*) AND TS = (removal OR clean* OR alternative OR laser OR “Er-YAG” OR “ER:YAG” OR GK-101* OR NMG OR papain OR “brix 3000” OR biosolv OR solvent* OR “carie-care” OR papacarie OR carisolv OR “*n*-monochloroglycine” OR caridex OR ozone OR “sono abrasion” OR “air abrasion” OR “air polishing” OR “photo-ablative” OR enzyme* OR chloramine OR “toluidine blue”)) AND DOCUMENT TYPES: (Article)
ClinicalTrials.gov	#1 Dental Caries
	Filters: Clinical trials, completed and with results.

**Table 4:** Chemomechanical caries removal systems versus control.

Author, Year, Study Design	Dentition, Groups (*n*)	Treatment Time (Mean ± SD)	Caries Removal	Anesthesia	Restoration	Patients Pain Perception/Behavior
Fure et al., 2000 [51] 1 or more txt/patient	PD C: Bur (26) T1: Carisolv (34)	C = 4.5 ± 2.0 T1 = 5.9 ± 2.2 (min), C < T1 *	Remaining caries: C = 1 Final cavity: C > T1	C = 6 T1 = 4	At 12 m follow-up Restorations lost: C = 2/24, T1 = 2/31; Secondary caries: C = 1/24; Marginal discoloration: C = 3/21, T1 = 4/29	Acceptance: 29/34 T1; T1: 64% did not find treatment times different and 23% found it faster than C; Better txt experience with T1 ***
Lozano-Chourio et al., 2006 [26] Cross-over	DD C: Bur (40) T1: Carisolv (40)	C = 2.47 ± 1.83 T1 = 7.51 ± 2.10 (min), C < T1 **	Final cavity dimension: C > T1 ***; Tissue removed: C > T1***	C = 2		Pain during txt: C = 40%, T1 = 17.5%; C > T1 *; More positive behavior in T1 **; Acceptance *: T1 = 71%
Pandit et al., 2007 [53] 1 or more txt/patient	DD C1: Excavator (50) C2: Bur (50) T1: Carisolv (50)	C1 = 424.6 ± 224.8 s C2 = 257.4 ± 100.6 s T1 = 534.8 ± 227.3 s C2 < C1 < T1	Efficacy C2 > C1, T1			VAS and VPS: C2 > C1 > T1
Subramaniam et al., 2008 [29] Split-mouth	DD C: Bur (20) T1: Carisolv (20)					
Mizuno et al., 2011 [30] Split-mouth	DD C: Excavator (8) T1: Papacarie (8)				At 6 m follow-up Success: C = 5/6, T = 6/6	
Anegundi et al., 2012 [12] Not clear	DD C: Bur (30) T1: Papacarie (30)	C = 280.89 s T1 = 1077.60 s C < T1 **	Final cavity dimension: C > T1 ***			No pain: C = 50%, T1 = 86.5%; Slight pain: C = 46.7%, T1 = 10%; Unspecified pain: C = 3.3%, T1 = 3.3%; More pain induced by C **; Acceptance: C = 36.7% and T1 = 60%
Rajakumar et al., 2013 [35] Split-mouth	DD C1: Excavator (20) C2: Bur (20) T1: Carie care (20)	C1 = 44.43 ± 5.05 s C2 = 30.39 ± 8.95 s T1 = 96.45 ± 10.99 s C2 < C1 < T1 ***	Efficacy C2 > T1 > C1			VPS and WBFPS: C1 > C2 > T1 **
Motta et al., 2013 [34] Split-mouth	DD C: Bur (20) T1: Papacarie (20)			C = 1		Pain: C > T1 *
Matsumoto et al., 2013 [33] Split mouth	DD C: Bur (20) T1: Papacarie Duo (20)	C = 1.43 ± 1.18 T1 = 2.45 ± 0.48 (min), C < T1		C = 1	At 30 d follow-up Restorations success: C∼T1 (*p* = 0.713)	Pain experience C∼T1 (*p* = 0.585)
Soni et al., 2014 [48] 1 txt/patient	DD and PD C1: Bur (30) C2: Excavator (30) C3: Polymer burs (30) T1: Carisolv (30)	C1 = 232 ± 21.99 s C2 = 480 ± 33.38 s C3 = 354 ± 23.21 s T1 = 697 ± 30.17 s C1 < C3 < C2 < T1 (intergroup comparison *)	Efficacy (dental explorer): C1 > C3 > T1 > C2. Efficacy (caries detector dye): C1 > T1 > C3 > C2. Intergroup comparison *: C1/C2, C2/T1, C2/C3			VAS and VPS: C1 > C2 > C3 > T1 (intergroup comparison *, except C3/T1)
Hegde et al., 2014 [36] Split-mouth	DD and PD C: Bur (16 each) T1: Carie-care (16 each)	DD C = 4.9; T1 = 7.0 (min), C < T1 PD C = 7.5; T1 = 10.4 (min), C < T1			At 12 m follow-up All restorations intact; amalgam restorations with better retention than Ketac molar. Ketac molar with more success in T1	T1 was more pleasant and accepted
Motta et al., 2014 [37] Split-mouth	DD C: Bur (20) T1: Papacarie (20)	C = 3.30 ± 2.03 T1 = 4.66 ± 3.13 (min), C < T1		C = 1	Success At 1 m follow-up: C = 100%, T1 = 100%; At 6 m follow-up: C = 90%, T1 = 95%; At 18 m follow-up: C = 80%, T1 = 95%	
Motta et al., 2014 [38] Split-mouth	DD C: Bur (20) T1: Papacarie (20)					
Goyal et al., 2015 [27] Cross-over	DD C: Bur (25) T1: Papacarie (25)	C = 6.99 ± 1.7 T1 = 7.41 ± 1.62 (min), C < T1				Mean pulse rate: C raised but reduced after txt; T1 reduced during and after txt ***; WBFPS: C raised during and after txt; T1 reduced **; Acceptance: C = 2/25, T1 = 20/25
Khalek et al., 2017 [45] Two parallel-arms	DD C: Excavator (25) T1: Papacarie (25)	C = 4.8 ± 1.0 T1= 5.8 ± 1.2 (min), C < T1*				Signs of pain/discomfort: Sound: C > T ****; Eye: C > T **; Motor: C > T ***
Bottega et al., 2018 [59] Not clear	DD and PD C: Bur (12) T1: Papacarie (12)	C = 10.5, T1 = 12.5 (min, median) C < T1			At 30 d follow-up T1 = 1/26 lost restorations	Heart rate rise during txt, maintaining after txt in C and T1
Ismail et al., 2019 [43] Split mouth	PD C: Bur (30) T1: Brix 3000 (30)	C = 23.53 ± 6.04 T1 = 37.67 ± 10.21 (min), C < T1 ***				Sound, eye and motor signs ****: Comfort: C = 3.3%, T1 = 30%; Mild discomfort: C = 6.6%, T1 = 50%; Moderately painful: C = 70%, T1 = 20%; Painful: C= 20%
Sontakke et al., 2019 [50] 1 txt/patient	PD C: Bur (30) T1: Carie-care (30)	C = 11.5 ± 2.83 T1 = 18.9 ± 3.78 (min), C < T1 ****				Pain: C > T1 ****

C: control group; d: day(s); DD: deciduous dentition; PD: permanent dentition; m: month(s); min: minutes; s: seconds; SD: standard deviation; T: test group; txt: treatment; VAS: visual analogue scale; VPS: verbal pain scale; WBFPS: Wong Baker Facial Pain Scale; Levels of significance. *: *p* < 0.05, **: *p* < 0.01, *** *p* < 0.001, **** *p* < 0.0001.

**Table 5:** Laser caries removal systems versus control.

Author, Year, Study Design	Dentition, Groups (*n*)	Treatment Time (Mean ± SD)	Caries Removal	Anesthesia	Restoration	Patients Pain Perception/Behavior
Hadley et al., 2000 [52] 1 or more txt/patient	PD C: Bur (66) T1: Er,Cr:YSGG laser (66)				At 30 d follow-up C: 1/66 restorations with partial loss; At 6 m follow-up C/T1: 66/66 restorations with no secondary caries	Discomfort: during txt C = 12.1%, T1 = 1.5%, C > T1 **; after txt C = 9.1%, T1 = 1.5%, C > T1 *
DenBesten et al., 2000 [46] 1 txt/patient	DD and PD C: Bur (41) T1: Er:YAG laser (82)		T1: txt energy for DD < PD ***	C = 25% T1 = 7%		Without discomfort (without anesthesia): C = 50%, T1 = 100%
DenBesten et al., 2001 [47] 1 txt/patient	C: Bur (42) T1: Er:YAG laser (82)	C = 6.6 T1 = 7.7 (min), C < T1	T1: txt energy for DD < PD *; energy for cavity preparation < caries removal *	C = 11/42 T1 = 4/82 C > T1 **		Without discomfort (without anesthesia: C = 31, T1 = 78): C = 50%, T1 = 100%
Liu et al., 2006 [28] Split-mouth	DD and PD C: Bur (40) T1: Er:YAG laser (40)	C= 41.3 ± 38.3 s T1= 97.0 ± 49.7 s C < T1 ***				More body movements during C ***; No pain: C = 17.5%, T1 = 82.5%; Mild pain: C = 40%, T1 = 12.5%; Moderate pain: C = 27.5%, T1 = 2.5%; Severe pain: C = 15%, T1 = 2.5%
Hjertton et al., 2013 [32] Split-mouth	PD C: Bur (35) T1: Er:YAG laser (35)	C = 16 ± 4 s T1= 59 ± 41 s C < T1 ***				Mean pulse raise during C and reduction during T1 *; Smell perception, comfort, acceptance: C < T1 **; Sound perception: C > T1 **
Eren et al., 2013 [31] Split-mouth	PD C: Bur (10) T1: Er,Cr:YSGG laser (10)	C = 3.7 T1 = 7.4 (min), C < T1				Pain: C > T1 Comfort: C < T1
Zhegova et al., 2014 [39] Split-mouth	PD C: Bur (44) T1: Er:YAG laser (44)					No pain: C = 18.18%, T1 = 79.55%, C < T1 *; Slight discomfort: C = 9.09%, T1 = 11.36%; Mild pain: C = 20.45, T1 = 9.09%; Moderate pain: C = 43.18%; Severe pain: C = 9.09%; Acceptance: C = 2/44, T1 = 38/44
Belcheva et al., 2014 [57] Not clear	DD and PD C: Bur (NR) T1: Er:YAG laser (NR)					Anxiety factors Vibration: C > T1 ***; Smell: C < T1 ***; Taste: C < T1 *
Belcheva et al., 2014 [58] Not clear	C: Bur (NR) T1: Er:YAG laser (NR)					Mild pain: C = 40%, T1 = 71.10%; Moderate pain: C = 42.20%, T1 = 26.70%; Severe pain: C = 17.80%, T1 = 2.20%; Pain: C > T1 **
Valério et al., 2015 [42] Split-mouth	DD C: Bur (29) T1: Er:YAG laser (29)	C= 54.96 ± 5.64 s T = 110.24 ± 9.83 s C < T1 *	Efficacy C∼T1, on pulpal walls *; C more efficient on the remaining walls ****		At 7 d and 12 m follow-up No differences	
Zhegova et al., 2015 [40] Split-mouth	PD C: Bur (58) T1: Er:YAG laser (58)				At 12 m follow-up Small marginal loss: C = 2/58; T1 = 3/58; At 18 m follow-up Small marginal loss: C/T1 = 2/58; small multiple losses or marginal discoloration: C/T1: 1/58; At 24 m follow-up Small marginal loss: C = 3/58, T1 = 2/58; small multiple losses or marginal discoloration: C = 1/58, T1 = 2/58	
Sarmadi et al., 2018 [10] Split-mouth	PD C: Bur (28) T1: Er:YAG laser (28)	C = 4.3 T1 = 13.2 (min), C < T1 ****		C = 15/28 T1 = 10/28	At 6 m follow-up Small marginal loss: C = 2/26; Slight discoloration: C= 1/26; Initial secondary caries: C = 2/26; At 12 m follow-up Small marginal loss: C/T1 = 1/25; Dentinal exposure: C = 1/25; Slight discoloration: C = 1/25; Initial secondary caries: T1 = 1/25; Secondary caries: C/T1 = 2/25; At 24 m follow-up Small marginal loss: C = 1/20; Secondary caries: T1 = 2/20	Discomfort after txt Immediate: C∼T1; At 7 d–C > T1 *; At 6 m–C > T1 ***; At 12 m and 24 m–C > T1 **; Acceptance: At 7 d–C < T1 ***; At 6 m, 12 m and 24 m–C < T1 **
Polizeli et al., 2019 [44] Split-mouth	DD C: Bur (20) T1: Er:YAG laser (20)				At 12 m follow-up C = 1/20 restorations were lost; Replicate SEM analysis Baseline∼6 m; after 12 m (gaps): C = 20%, T1 = 10%	Raise of salivary cortisol levels during txt and C∼T1

C: control group; d: day(s); DD: deciduous dentition; Er:YAG: Erbium-doped Yttrium Aluminium Garnet; Er,Cr:YSGG: Erbium, Chromium-doped Yttrium, Scandium, Gallium and Garnet; PD: permanent dentition; m: month(s); min: minutes; NR: not reported; s: seconds; SD: standard deviation; SEM: scanning electronic microscope; T: test group; txt: treatment; Levels of significance. *: *p* < 0.05, **: *p* < 0.01, *** *p* < 0.001, **** *p* < 0.0001.

**Table 6:** Studies with multiple comparisons.

Author, Year, Study Design	Dentition, Groups (*n*)	Treatment Time (Mean ± SD)	Caries Removal	Patients Pain Perception/Behavior
Kochhar et al., 2011 [54] 1 or more txt/patient	DD C1: Excavator (30) C2: Bur (30) T1: Carisolv (30) T2: Papacarie (30)	C1 = 535.83 ± 232.14 s C2 = 261.70 ± 86.06 s T1 = 723.73 ± 179.48 s T2 = 590.80 ± 187.00 s C2 < C1 < T2 < T1 (intergroup comparison *, except C1/T2)	Remaining caries: C2 < T2 < T1 < C1 (intergroup comparison *, except T2/C2 and T2/T1)	VAS and VPS: intergroup comparison *, except C1/C2 on VPS; VAS and VPS: C2 > C1 > T1 > T2
Bohari et al., 2012 [56] Not clear	DD C: Bur (30) T1: Carisolv (30) T2: Papacarie (30) T3: Er:YAG laser (30)	C = 206.7 ± 22.1 s T1 = 474.7 ± 43.0 s T2 = 471.3 ± 68.2 s T3 = 359.9 ± 59.9 s (intergroup comparison ***, except T1/T2)	Change in DIAGNOdent values: C = 92.9 ± 9.2%; T1 = 87.7 ± 6.4%; T2 = 88.9 ± 6.0%; T3 = 93.9 ± 4.9%; (intergroup comparison *, for C/T1 and T1/T3, and **, for C/T2 and T2/T3)	FLACCS: C was more painful and uncomfortable (intergroup comparison of T1, T2, T3/C ***)
Ammari et al., 2014 [15] 1 or more txt/patient	DD C: Excavator (22) T1: Papacarie (25) T2: Carisolv (27)			
Hegde et al., 2016 [41] Split-mouth	DD C: Bur (50) T1: Carisolv (50) T2: Papacarie (50)	C = 61.34 ± 30.43 s T1 = 341.21 ± 110.93 s T2 = 322.49 ± 91.24 s C< T1, T2 ***	Efficacy: C *** > T1, T2; T1 < T2 **	Pain perception: C > T1 > T2, C > T1 *, C > T2 **; Worsening of behavior during C **; Acceptance *: C = 24%, T1 = 26% and T2 = 48%
Alkhouli et al., 2020 [49] 1 txt/patient	DD C: Bur (10) T1: 2.25% sodium hypochlorite gel (12) T2: Brix 3000 (10)	C= 1.60 ± 0.843 T1= 6.40 ± 1.838 T2= 5 ± 1.595 (min), C < T1 ***, C < T2 **		WBFPS: C > T1 ** and C > T2 ***

C: control group; DD: deciduous dentition; Er:YAG: Erbium-doped Yttrium Aluminium Garnet; FLACCS: face, leg, activity, cry, consolability scale; min: minutes; s: seconds; SD: standard deviation; T: test group; txt: treatment; VAS: visual analogue scale; VPS: verbal pain scale; WBFPS: Wong Baker Facial Pain Scale; Levels of significance. *: *p* < 0.05, **: *p* < 0.01, ***: *p* < 0.001.

**Table 7:** Summary findings.

Outcomes	Mechanical	Chemomechanical	Laser	Vector System
Treatment time	Conventional methods for caries removal determine faster treatment interventions [10,12,15,26,27,28,29,30,31,32,33,34,35,36,37,38,39,40,41,42,43,44,45,46,47,48,49,50,51,52,53,54,55,56,57,58,59]	Brix 3000, Carie-care, Carisolv and Papacarie led to longer interventions than conventional treatments [12,26,43,45,48,49,51]	Treatment with the Er:YAG laser system increased treatment time [10,28,32,42], while treatment with the Er,Cr:YSGG did not [31]	The use of VS increased treatment time [55]
		The use of Carisolv and Papacarie increases treatment time compared to the use of the Er:YAG laser system, and Papacarie determines faster interventions than Carisolv [56]		
Caries removal efficacy	Mechanical caries removal produces wider free-caries cavities [12,26]	Papacarie tends to be more effective in caries removal than Carisolv [41,54]	Treatment in the deciduous dentition requires less energy than treatment in the permanent dentition [46,47]	
		Treatment with Er:YAG laser showed the highest percentage of DIAGNOdent value change, compared to Carisolv and Papacarie, and Papacarie showed higher values change than Carisolv [56]		
Anesthesia	Patients requested anesthesia more often when treated with mechanical methods [10,26,33,34,37,46,47,51]	Treatment with chemomechanical or laser methods was performed with less requests for anesthesia [10,26,33,34,37,46,47,51]		
CFU count	Mechanical rotary methods are effective in reducing the cariogenic flora [12,15,27,29,38,43]	Chemomechanical methods are effective in reducing the cariogenic flora [12,15,27,29,38,43] and Brix 3000 and Papacarie may achieve higher reductions than mechanical methods [12,28,43]		
Restorations	The restorations performance did not differ according to the method for caries removal [10,30,33,36,37,40,42,44,51,52,59]			
Patients pain perception/behavior	Mechanical caries removal is associated with pain, more movements during treatment and is less preferred regarding future treatments [10,12,26,27,28,32,33,34,35,36,39,41,43,45,48,49,50,51,52,53,54,55,56,57,58,59]	All alternative methods induce less pain and discomfort during treatment. However, smell and taste may be appointed as negative aspects of these approaches [10,12,26,27,28,32,33,34,35,36,39,41,43,45,48,49,50,51,52,53,54,55,56,57,58,59];Alternative methods are widely accepted and preferred for future treatments, and Papacarie showed higher preference, mostly because of less induced pain [12,27,33,34,41,45,54,56,59]		

CFU: colony forming units; Er:YAG: Erbium-doped Yttrium Aluminium Garnet; Er,Cr:YSGG: Erbium, Chromium-doped Yttrium, Scandium, Gallium and Garnet.

## Appendix A

**Table A1:** Evaluation of quality assessment of the randomized clinical trials included in this systematic review.

1	2	3	4	5	6	7	
?	?	?	?	+	+	?	Chomyszyn-Gajewska et al., 2006 [55]
?	?	?	?	+	+	?	Fure et al., 2000 [51]
+	?	?	+	+	+	?	Lozano-Chourio et al., 2006 [26]
?	?	?	?	+	+	?	Pandit et al., 2007 [53]
?	?	?	?	+	+	?	Subramaniam et al., 2008 [29]
?	?	?	?	+	+	?	Mizuno et al., 2011 [30]
?	?	?	?	+	+	?	Anegundi et al., 2012 [12]
?	?	?	?	+	+	?	Rajakumar et al., 2013 [35]
+	?	?	+	+	+	?	Motta et al., 2013 [34]
+	?	?	?	+	+	?	Matsumoto et al., 2013 [33]
+	?	?	?	+	+	?	Soni et al., 2014 [48]
	?	?	?	+	+	?	Hegde et al., 2014 [36]
+	?	?	+	+	+	?	Motta et al., 2014 [37]
+	?	?	?	+	+	?	Motta et al., 2014 [38]
?	?	?	?	+	+	?	Goyal et al., 2015 [27]
+	+	?	?	+	+	?	Khalek et al., 2017 [45]
+	?	?	+	+	+	?	Bottega et al., 2018 [59]
?	?	?	?	+	+	?	Ismail et al., 2019 [43]
?	?	?	+	+	+	?	Sontakke et al., 2019 [50]
+	?	?	+	+	+	?	Hadley et al., 2000 [52]
?	?	?	+	?	?	?	DenBesten et al., 2000 [46]
?	?	?	+	?	?	?	DenBesten et al., 2001 [47]
+	?	?	?	+	+	?	Liu et al., 2006 [28]
?	?	?	?	+	+	?	Hjertton et al., 2013 [32]
x	?	?	?	+	+	?	Eren et al., 2013 [31]
?	?	?	?	+	+	?	Zhegova et al., 2014 [39]
?	?	?	?	+	+	?	Belcheva et al., 2014 [57]
?	?	?	?	+	+	?	Belcheva et al., 2014 [58]
+	?	?	+	+	+	?	Valério et al., 2015 [42]
?	?	?	?	+	+	?	Zhegova et al., 2015 [40]
+	+	?	+	+	+	?	Sarmadi et al., 2018 [10]
+	?	?	?	+	+	?	Polizeli et al., 2019 [44]
?	?	?	?	+	+	?	Kochhar et al., 2011 [54]
?	?	?	?	+	+	?	Bohari et al., 2012 [56]
?	?	?	?	+	+	?	Ammari et al., 2014 [15]
+	?	?	?	+	+	?	Hegde et al., 2016 [41]
+	?	?	?	+	+	?	Alkhouli et al., 2020 [49]

1: Random sequence generation (selection bias); 2: Allocation concealment (selection bias); 3: Blinding of participants and personnel (performance bias); 4: Blinding of outcome assessment (detection bias); 5: Incomplete reporting data (attrition bias), 6: Selective reporting (reporting bias); 7: Other bias.
